# Towards the differential diagnosis of prostate cancer by the pre-treatment of human urine using ionic liquids

**DOI:** 10.1038/s41598-020-71925-8

**Published:** 2020-09-10

**Authors:** Matheus M. Pereira, João D. Calixto, Ana C. A. Sousa, Bruno J. Pereira, Álvaro S. Lima, João A. P. Coutinho, Mara G. Freire

**Affiliations:** 1 Department of Chemistry, CICECO-Aveiro Institute of Materials, University of Aveiro, 3810-193 Aveiro, Portugal; 2 Faculty of Health Sciences, University of Beira Interior, 6200-506 Covilhã, Portugal; 3 Instituto Português de Oncologia de Coimbra Francisco Gentil, 3000-075 Coimbra, Portugal; 4 Programa de Pós-Graduação Em Engenharia de Processos, Universidade Tiradentes, Farolândia, Aracaju, SE CEP 49032-490 Brazil

**Keywords:** Proteins, Diseases, Chemistry, Engineering, Biomarkers, Diagnostic markers

## Abstract

Prostate specific antigen (PSA) is the most widely used clinical biomarker for the diagnosis and monitoring of prostate cancer. Most available techniques for PSA quantification in human fluids require extensive sample processing and expensive immunoassays that are often unavailable in developing countries. The quantification of PSA in serum is the most common practice; however, PSA is also present in human urine, although less used in diagnosis. Herein we demonstrate the use of ionic-liquid-based aqueous biphasic systems (IL-based ABS) as effective pre-treatment strategies of human urine, allowing the PSA detection and quantification by more expedite equipment in a non-invasive matrix. If properly designed, IL-based ABS afford the simultaneous extraction and concentration of PSA (at least up to 250-fold) in the IL-rich phase. The best ABS not only allow to concentrate PSA but also other forms of PSA, which can be additionally quantified, paving the way to their use in differential prostate cancer diagnosis.

## Introduction

Prostate cancer (PCa) is the second most common type of cancer in men and the fifth leading cause of cancer death worldwide, with the highest mortality rates found in the Caribbean and Southern and Middle Africa^1^. Age is considered the most significant risk factor, with 97% of PCa cases occurring in men over 50 years old^2,3^. In a current aging society, the incidence of PCa is expected to increase in the coming years^1,4^. Therefore, effective diagnosis and treatment monitoring are of utmost relevance to decrease the health and economic burdens associated to the increasing incidence of PCa. The clinical “gold standard” biomarker for PCa is the prostate specific antigen (PSA). This glycoprotein, produced by the prostate gland, is usually found at higher or lower levels, respectively, in the blood or urine of prostate cancer patients. PSA was approved by the Food and Drug Administration (FDA) in 1986 as a biomarker, and since then has been used for the early stage diagnosis of PCa. Despite some controversies^5^ and efforts to find alternative biomarkers and novel detection technologies^6^, PSA is still the most reliable clinical biomarker currently used in PCa diagnosis^7^.

There are several commercial techniques available for the PSA quantification in blood and urine samples; however, they are generally based on expensive and time-consuming immunoassays and require an extensive sample processing or pre-treatment to decrease interferences^8–11^. Such degree of complexity and associated high cost of the analysis is not feasible to apply in all laboratories, particularly in those from developing nations where PCa mortality is higher^12^. In addition to blood/serum, PSA can be found in other human fluids, including urine, a non-invasive matrix that additionally does not require trained personnel for samples collection. Currently, urinary PSA levels are used in the differential diagnosis of PCa, particularly when serum PSA levels are between 2.5 and 10 ng mL^−1^, the so-called “grey zone” where the differentiation between PCa and benign prostate hyperplasia (BPH) is difficult^13^. Based on the exposed, the development of pre-treatment strategies of human biological fluids that could allow the accurate evaluation of PSA levels in serum and/or urine using cost-effective and expedite methods is highly desirable for PCa screening.

Herein we show the applicability of ionic-liquid-based aqueous biphasic systems (IL-based ABS) to simultaneously extract and concentrate PSA from human urine samples to improve PCa diagnosis and monitoring. ABS are formed by two compounds (e.g. two polymers, a polymer and a salt, or two salts) that undergo phase separation when dissolved in water above given concentrations. Although most ABS are considered amenable media for proteins due to their high water content, polymer-based ABS display a limited polarity range between the two phases, hampering high extraction efficiencies to be achieved in one-step. Previous works reported on the use of ABS composed of two polymers, namely dextran and ficoll, to extract PSA^14^; nevertheless, these systems do not allow the complete extraction and concentration of PSA in one phase. Accordingly, subsequent studies^15,16^ focused on the quantification of PSA isoforms and determination of their partition coefficients between the ABS coexisting phases. Overall, in all studies reported so far, it was not possible to completely extract PSA to a given phase, thus preventing the use of ABS in pre-treatment strategies of human fluids as proposed in the current work. Furthermore, due to the low amounts of the target biomarker, the quantification of PSA in these works^15,16^ was performed by immunoassays, an expensive technique often unavailable in developing countries.

We hereby demonstrate the use of ILs as phase-forming components of ABS, which due to their designer solvents ability permit to broaden the phases’ polarities^17,18^ and to improve the systems selectivity to PSA. IL-based ABS, if properly designed, are able to completely extract target proteins and keep their native form and stability in an aqueous-rich media. Accordingly, IL-based systems can be envisioned as pre-treatment strategies of human urine to improve the PCa diagnosis and monitoring by the PSA accurate identification and quantification, while minimizing the influence of other proteins and metabolites. Most of the ILs investigated for the formation of IL-based ABS to carry out the extraction of proteins display however some biocompatibility concerns^17^ and do not allow the pH control, a crucial request when aiming the total recovery and the stability maintenance of a protein such as PSA. To simultaneously increase the biocompatible nature of ILs and to control the pH without the need of adding external buffers, ILs with self-buffering characteristics derived from biological Good’s buffers (GB-ILs)^19,20^ can be considered promising alternatives, being studied in this work.

## Results

### Ionic-liquid-based aqueous biphasic systems

**Figure 1:**
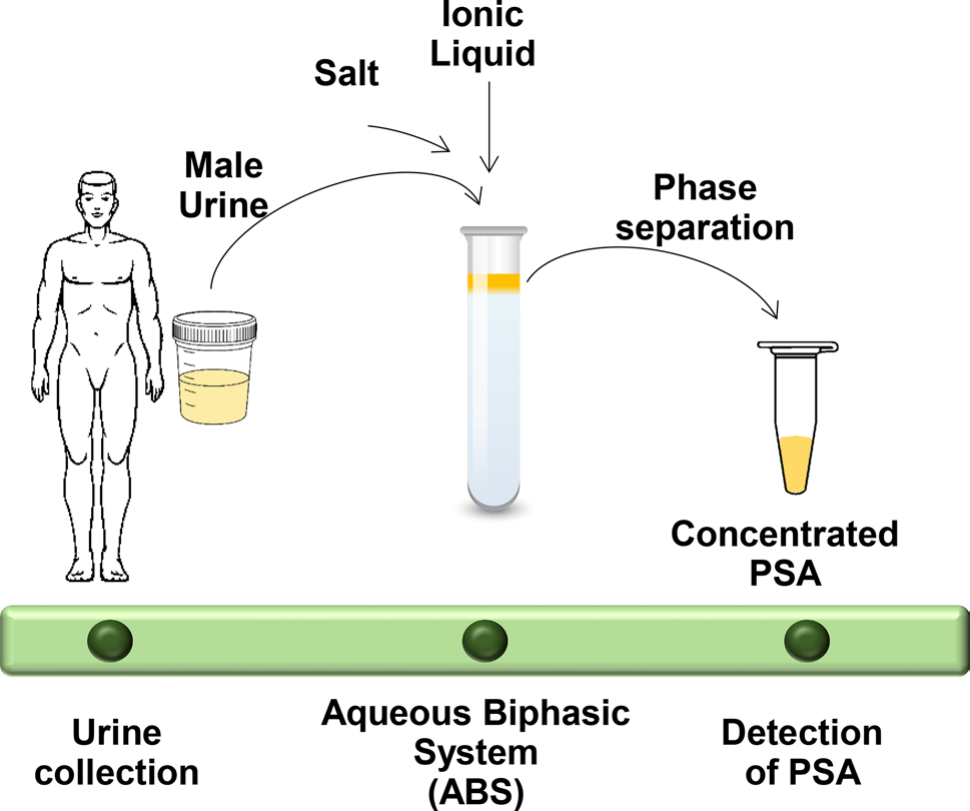
Schematic representation of the goal and achievements of this work.

ABS composed of GB-ILs and a biodegradable organic salt, K_3_C_6_H_5_O_7_, are here demonstrated to act as effective pre-treatment strategies of human urine by simultaneously extracting and concentrating PSA to the IL-rich phase. If the IL is properly designed, no losses of protein occur, thus not leading to false/positive results and not compromising the diagnosis/prognosis. Moreover, the concentration factor should occur in an order of magnitude that the cancer biomarker at the IL-rich phase is detectable using simpler and less laborious techniques, being in this work quantified by size exclusion high performance liquid chromatography (SE-HPLC) instead of immunoassays. A schematic representation of the goal and achievements of this work is provided in Fig. 1. The ionic liquids investigated are formed by the tetrabutylphosphonium ([P_4444_]^+^) cation and GB-derived anions, which were synthesized and characterized by us according to established protocols^18–20^. The following ILs were investigated: tetrabutylphosphonium 2-(N-morpholino)ethanesulfonate ([P_4444_][MES]), tetrabutylphosphonium 2-[[1,3-dihydroxy-2-(hydroxymethyl)propan-2-yl]amino]ethanesulfonate ([P_4444_][TES]), tetrabutylphosphonium 2-(cyxlohexylamino)ethanesulfonate ([P_4444_][CHES]), tetrabutylphosphonium 2-[4-(2-hydroxyethyl)piperazin-1-yl]ethanesulfonate ([P_4444_][HEPES]) and tetrabutylphosphonium N-2(2-hydroxy-1,1-bis(hydroxymethyl)ethyl)glycine ([P_4444_][Tricine]). Their characterization and chemical structures are given in the Supporting Information.

**Figure 2:**
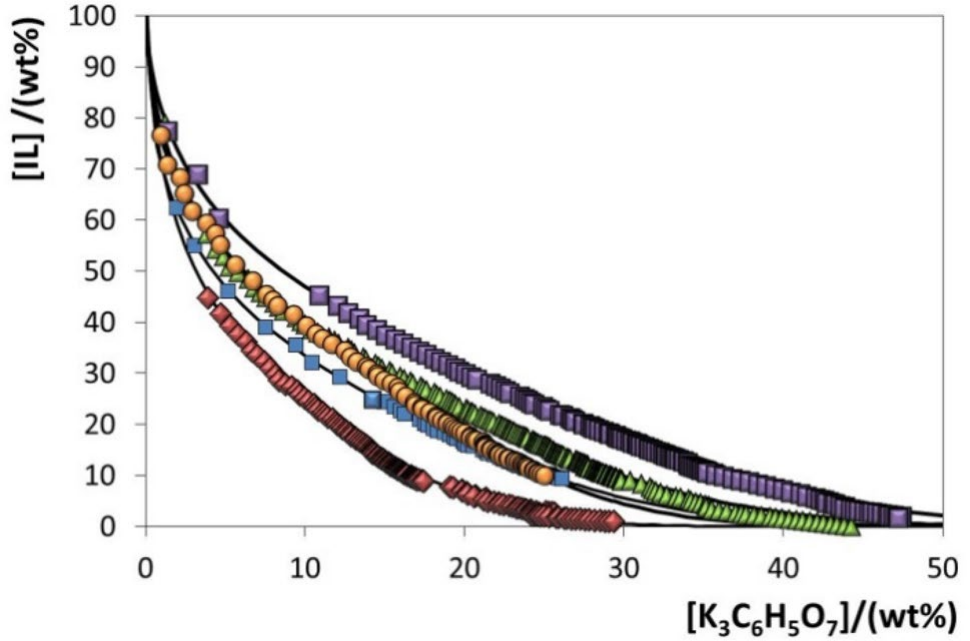
Phase diagrams for the systems composed of GB-IL + K_3_C_6_H_5_O_7_ + H_2_O at 25 °C: [P_4444_][Tricine] (purple square); [P_4444_][HEPES] (green triangle); [P_4444_][TES] (orange circle); [P_4444_][MES] (blue square); [P_4444_][CHES] (red diamond); and fitted binodal data (straight line).

In order to identify the mixture compositions required to perform a liquid–liquid extraction by means of ABS, the respective ternary phase diagrams at 25 °C and at atmospheric pressure were initially determined, being shown in Fig. 2. The respective experimental data are given in the Supporting Information. In all ABS, the biphasic region is positioned above the solubility curve while the monophasic region is located below. Diagrams with the largest area above the binodal curve have a higher ability to form two phases, i.e. the IL is more easily salted-out by the salt. The citrate-based salt used is composed of a trivalent charged anion and is a strong salting-out species according to the Hofmeister series^21^. Therefore, it has a high affinity for water and there is the preferential exclusion of the IL ions from the aqueous solution, promoting the two-phase separation.

At 10 wt% of K_3_C_6_H_5_O_7_, the ability of GB-ILs to create ABS follows the order: [P_4444_][CHES] > [P_4444_][MES] > [P_4444_][HEPES] ≈ [P_4444_][TES] > [P_4444_][Tricine]. Since the salt, temperature, pressure and pH are constant in the studied two-phase systems, the ABS formation ability is a result of the IL nature. It was already demonstrated that [P_4444_]-based ILs display a high ability to undergo phase separation in presence of salts when compared to ammonium-, pyridinium- and imidazolium-based counterparts^22^. [P_4444_]-based salts have highly shielded charges, located mostly on the heteroatom surrounded by four alkyl chains, being easily salted-out from aqueous media. On the other hand, anions have a higher aptitude for creating hydration complexes because they are more polarizable and present a more diffuse valence electronic distribution^23^. IL anions with lower ability to establish hydrogen bonds and to create hydration complexes are more easily salted-out^23^, a trend that can be correlated with the respective octanol–water partition coefficients (*K*_*ow*_). The higher the log(*K*_*ow*_) value the lower affinity of the anion for the water-rich phase and to be hydrated. Accordingly, the ILs composed of anions with –OH groups and lower values of log(*K*_*ow*_) ([Tricine]: − 5.25; [TES]: − 4.48; [HEPES]: − 3.11)^24^ are those that correspond to ILs that present a solubility curve more distant from the axis in Fig. 2, requiring higher amounts of salt for phase separation. On the other hand, GBs with no –OH groups and with higher log(*K*_*ow*_) values ([MES]: − 2.48; [CHES]: − 0.59)^24^ have a lower aptitude to hydrogen-bond with water and a higher capacity for liquid–liquid demixing in ABS, as shown in Fig. 2.

### Extraction and concentration of prostate specific antigen (PSA)

After addressing the mixture compositions required to create ABS that could act as pre-treatment strategies of human fluids by liquid–liquid extraction, these systems were evaluated in what concerns their ability to extract PSA. This initial screening on the ABS extraction and recovery performance was carried out with commercial PSA in aqueous solution at a concentration ca. 50 ng mL^−1^. Two mixture compositions were studied: 30 wt% of IL + 30 wt% of salt and 30 wt% of IL and 40 wt% of salt. In this step, the quantification of PSA was carried out by a Blitz system that uses immobilized anti-PSA (experimental details are given in the Supporting Information), allowing the direct quantification of biologically active PSA and to infer the protein loss of stability. It should be remarked that, at this concentration level no PSA can be detected by SE-HPLC if a concentration step is not carried out, as shown below. The extraction efficiencies of PSA in all ABS and mixture compositions evaluated correspond to 100%, meaning that PSA is only present in the IL-rich phase (no PSA detected at the salt-rich phase). However, not all systems allow the complete recovery of stable PSA into the IL-rich phase, with recovery efficiencies ranging between 7 and 99 (± 1) %. Detailed data are given in the Supporting Information. Overall, the extent of protein loss follows the ILs rank in ABS formation (cf. Fig. 2), in which the most hydrophobic ILs, namely [P_4444_][CHES] and [P_4444_][MES], are those that do not lead to protein losses. Accordingly, ABS formed by [P_4444_][CHES] and [P_4444_][MES] were selected to investigate the use of ABS as simultaneous extraction and concentration steps, ultimately allowing their use in the pre-treatment of human urine samples.

**Figure 3:**
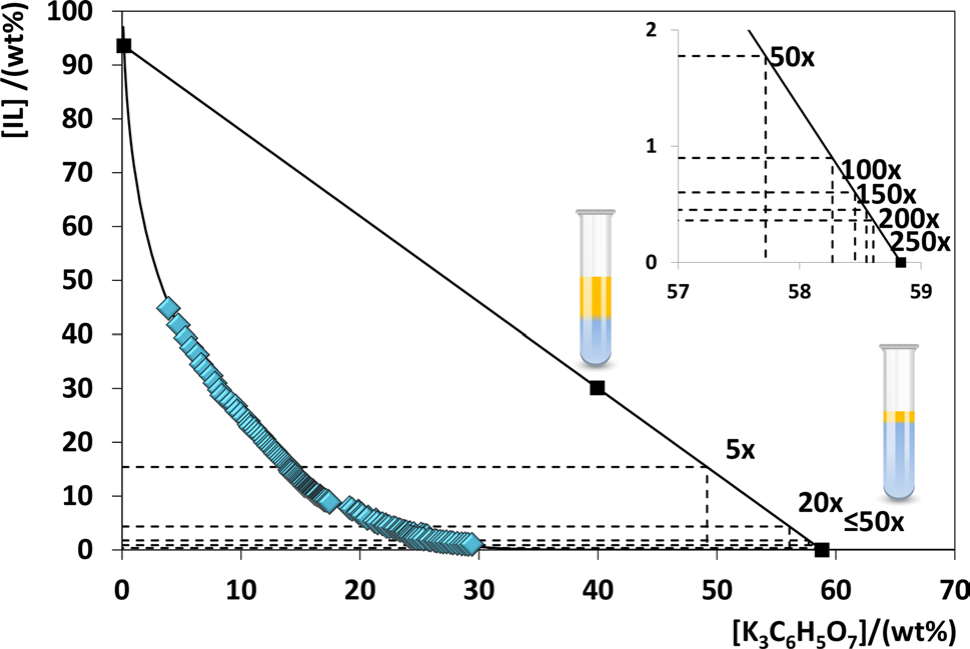
Schematic representation of the lever-arm rule and achievable CFs for the system composed of [P_4444_][CHES] + K_3_C_6_H_5_O_7_ + H_2_O at 25 °C, and adjusted binodal data.

By the characterization of the ABS phase diagrams, including a series of tie-lines that give the composition of each phase for a given mixture compositions, it is possible to prepare mixtures with known compositions and defined phases’ weights or volumes. Along the same tie-line, by decreasing the amount of IL added and increasing the amount of salt, the IL-rich phase has a continuously reduced volume with a concomitant increase of the concentration factor (CF), as depicted in Fig. 3. Detailed data on the mixtures compositions required to achieve the CF of 250 are given in the Supporting Information. CFs up to 250-fold are possible to achieve with the two IL-based ABS investigated. By using aqueous solutions and human urine spiked with commercial PSA at 150 ng∙mL^−1^ (threshold value proposed for PSA in urine)^13^ in the composition of these ABS and with the CF of 250, there is the decrease of the volume of the IL-rich phase that allows the PSA detection and quantification by an equipment with a higher detection limit, such as SE-HPLC, and as shown in the Supporting Information. The detailed extraction and recovery efficiencies of each system with the required CF of 250 are given in the Supporting Information. The experimental conditions required for the SE-HPLC analysis are also provided in the Supporting Information.

**Figure 4:**
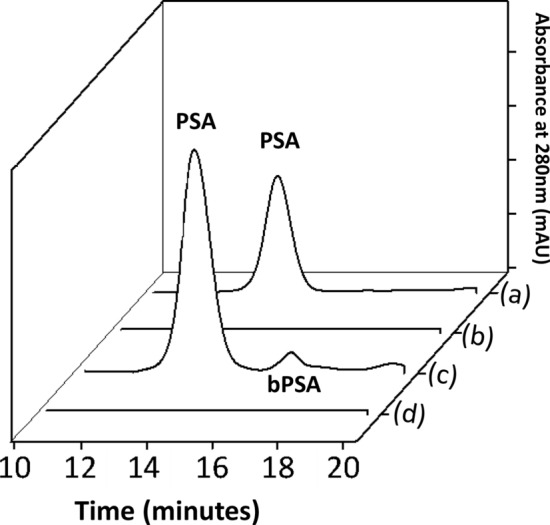
SE-HPLC profiles of (**a**) pure/commercial PSA in aqueous solution; (**b**) non-treated human urine; (**c**) IL-rich phase of pre-treated human urine; and (**d**) salt-rich phase of pre-treated human urine.

Based on the results of the previous screening, the ABS formed by [P_4444_][CHES] allowing a CF of 250 was applied to directly pre-treat human urine samples (with no spiked PSA). Figure 4 depicts the SE-HPLC chromatogram of an aqueous solution of pure/commercial PSA, with a retention time of 13.5 min under the operational conditions used, of the non-treated urine sample (where no PSA is detected) and of urine samples pre-treated with IL-based ABS.

The HPLC peak corresponding to PSA is not seen in the non-treated urine samples neither in the bottom (salt-rich) phase of the ABS. However, a clear peak in the IL-rich phase corresponding to PSA in the pre-treated urine samples is clearly seen and can be accurately quantified. Remarkably, the SE-HPLC proteins profile also reveals the presence of an additional protein, with a retention time between 16 and 17 min, only in the IL-rich phase of pre-treated human urine samples. An analysis of PSA by HIC-HPLC identified this peak as bPSA^25^. PSA is a glycoprotein that can be found in different forms; the levels of these different forms and their respective ratio can be used in PCa differential diagnosis^25^. Therefore, the best identified ABS not only allows to concentrate PSA, but also bPSA which can be additionally quantified. Based on these results, adequate IL-based ABS can be used to effectively pre-treat human urine samples, leading to the concentration and quantification of more than one form of PSA while paving the way to PCa differential cancer diagnosis and monitoring. The approach here proposed is different from that reported in previous studies dealing with ABS composed of two polymers^14–16^. Instead of analysing the partition behaviour of PSA and respective isoforms, and where the partition coefficient is directly used in diagnosis, in this work IL-based ABS are used as pre-treatment strategies of biological samples, allowing the quantification of cancer biomarkers by SE-HPLC and not by immunoassays as usually carried out^14–16^.

In summary, in this work we demonstrate the remarkable ability of IL-based ABS to act as pre-treatment strategies of human urine samples. If properly designed, IL-based ABS allow the complete extraction of PSA, with no protein losses, and a concentration factor up to 250-fold, allowing the identification and quantification of PSA and bPSA using less time-consuming and expensive techniques, particularly valuable in PCa diagnosis and monitoring in developing countries. Moreover, the demonstrated possibility of extracting and concentrating different PSA forms with IL-based ABS opens an exciting possibility for future research on the prostate cancer differential diagnosis, ultimately leading to a decrease on the health and economic burdens related with the increasing incidence of PCa.

## Methods

### Materials

Potassium citrate tribasic monohydrate (K_3_C_6_H_5_O_7_.H_2_O, purity ≥ 99 wt%) was obtained from Sigma-Aldrich Chemical Co. (USA). Prostate Specific Antigen from human semen (purity ≥ 95%) was obtained from Sigma-Aldrich Chemical Co. Methanol (purity > 99.9%) was acquired from Fisher Scientific. Acetonitrile (purity > 99.7%) was supplied from Lab-Scan. The buffers required for the ILs synthesis, namely *n*-cyclohexyl-2-aminoethanesulfonic acid (CHES, purity > 99 wt%), 4-(2-hydroxyethyl)-1-piperazineethanesulfonic acid (HEPES, purity > 99.5 wt%), 2-(N-morpholino)ethanesulfonic acid (MES, purity > 99 wt%), n-(tri(hydroxymethyl)methyl)glycine (Tricine, purity > 99 wt%) and 2-[[1,3-dihydroxy-2-(hydroxymethyl)propan-2-yl]amino]ethanesulfonic acid (TES, purity > 99 wt%) were purchased from Sigma-Aldrich Chemical Co. The cation precursor, tetrabutylphosphonium hydroxide ([P_4444_][OH], 40 wt% in H_2_O) was supplied by Sigma–Aldrich Chemical Co. Tetramethylsilane (TMS, purity > 99.9 wt%) and deuterium oxide (D_2_O purity > 99.9 wt%) were obtained from Sigma–Aldrich Chemical Co. Purified water passed through a reverse osmosis and a Milli-Q plus 185 water purifying system was used in all experiments. For the quantification of PSA using the Blitz Pro System, Super Streptavidin biosensors acquired from VWR were used.

### Biological samples

Urine samples were provided by a healthy male donor after signing an informed consent according to the Declaration of Helsinki. The collection of urine samples was approved by the Ethics Committee of Cova da Beira University Hospital Center (CE 41/2015). The collection of data complied with the General Data Protection Regulation (Reg. (EU) 2016/679 and all procedures were carried out in accordance with relevant guidelines and regulations.

### Synthesis and characterization of the Good buffers-ionic liquids (GB-ILs)

The GB-ILs ([P_4444_][MES], [P_4444_][TES], [P_4444_][CHES], [P_4444_][HEPES] and [P_4444_][Tricine]—chemical structures and complete descriptions given in in Supporting Information) were synthesized via neutralization of the base with the appropriate acid, according to previously reported protocols^26^. Briefly, a slightly excess of an equimolar aqueous solution (1:1.1) of buffer was added drop-wise to the tetrabutylphosphonium hydroxide solution. The mixture was stirred continuously for at least 12 h at room temperature (≈ 25 °C) to produce the IL and water as by-product. The mixture was then subjected to 50–60 °C under reduced pressure, resulting in a viscous liquid. A mixture of acetonitrile and methanol (1:1, v:v) was added and vigorously stirred at room temperature for 1 h. The solution was then filtered to remove any excess buffer. The organic solvents were evaporated and the GB-IL products dried under vacuum for 3 days at room temperature. The water content in each GB-IL was measured by Karl-Fischer (KF) titration, using a KF coulometer (Metrohm Ltd., model 831)—data given in the Supporting Information. The chemical structures and purity of the GB-ILs were confirmed by ^1^H and ^13^C NMR spectroscopy (Bruker AMX 300) operating at 300.13 and 75.47 MHz, respectively, whose spectra are given in the Supporting Information. Chemical shifts are expressed in δ (ppm) using tetramethylsilane (TMS) as internal reference and D_2_O as deuterated solvent. The ILs synthetized in this work showed high purity levels without signs of decomposition.

### Phase diagrams and tie-lines

To ascertain the mixture compositions required to form two-phase systems that can be used as extraction/concentration platforms, the binodal curve of each ABS were initially determined through the cloud point titration method^17^ at 25 °C (± 1 °C) and atmospheric pressure. Aqueous solutions of K_3_C_6_H_5_O_7_ at circa 60 wt % and aqueous solutions of the different ILs (≈ 80 wt%) were prepared and used for the determination of the binodal curves, followed by the drop-wise addition of water until the finding of the monophasic region. The opposite procedure also was carried out to better describe the binodal curves, particularly at the salt-rich region, which is of extreme relevance to deal with ABS as concentration strategies. The ternary system compositions were determined by weight quantification within ± 10^–4^ g. The experimental binodal curves at 25 °C were fitted by Eq. (1)^27^:

1 $$\left[ {IL} \right] = A{\text{ exp}}\left( {B\left[ {salt} \right]_{{}}^{0.5} } \right) - \left( {C\left[ {salt} \right]_{{}}^{3} } \right)$$

where [*IL*] and [*salt*] are the IL and the salt weight fraction percentages, respectively, and *A*, *B* and *C* are constants obtained by the regression of the experimental data.

Tie-lines (TLs), i.e. the composition of each phase for a given initial mixture composition at the biphasic region, were determined through the solution of the following system of four equations (Eqs. (2) to (5)) with four unknown values ([*IL*]_*IL*_, [*IL*]_*salt*_, [*salt*]_*IL*_, and [*salt*]_*salt*_):

2 $$\left[ {IL} \right]_{salt} = Aexp\left[ {\left( {B\left[ {salt} \right]_{IL}^{0.5} } \right) - \left( {C\left[ {salt} \right]_{IL}^{3} } \right)} \right]$$

3 $$\left[ {IL} \right]_{salt} = Aexp\left[ {\left( {B\left[ {salt} \right]_{salt}^{0.5} } \right) - \left( {C\left[ {salt} \right]_{salt}^{3} } \right)} \right]$$

4 $$\left[ {IL} \right]_{IL} = \frac{{\left[ {IL} \right]_{M} }}{\alpha } - \frac{1 - \alpha }{\alpha }\left[ {IL} \right]_{salt}$$

5 $$\left[ {salt} \right]_{IL} = \frac{{\left[ {salt} \right]_{M} }}{\alpha } - \frac{1 - \alpha }{\alpha }\left[ {salt} \right]_{salt}$$

where the subscripts *IL*, *salt* and *M* represent the top and the bottom phases and the mixture composition, respectively. The parameter α is the ratio between the weight of the top phase and the weight of the overall mixture. The respective tie-line lengths (TLLs) were determined by Eq. (6):

6 $$TLL = \sqrt {\left( {\left[ {salt} \right]_{IL} - \left[ {salt} \right]_{salt} } \right)^{2} + \left( {\left[ {IL} \right]_{IL} - \left[ {IL} \right]_{salt} } \right)^{2} }$$

### Extraction and quantification of prostate specific antigen (PSA) using IL-based ABS

An initial screening on the several ILs performance and mixture compositions was carried out, particularly to appraise the ABS ability to extract PSA from aqueous solutions without protein losses. To this end, different ABS, involving all the prepared ILs, and two mixture compositions (40 wt% salt + 30 wt% IL + 30 wt% H_2_O and 40 wt% salt + 40 wt% IL + 30 wt% H_2_O) were studied to evaluate the effect of the concentration of the phase-forming components. Aqueous solutions of PSA at concentrations circa 50 ng mL^−1^ were used as the “water” added to each ABS. The used PSA concentration was chosen after optimization using the BLItz Pro system. This type of quantification was chosen since it allows also to address the PSA stability and activity, where only active PSA binds with PSA-antibody. After, a careful separation of the phases was performed and the amount of PSA in each phase was quantified using the BLItz Pro system equipment, by an external standard calibration method using protein concentrations ranging from 1 to 100 ng mL^−1^. The anti-PSA solution was prepared in sample diluent buffer (PBS) at 100 µg mL^−1^. Anti-PSA was immobilized onto streptavidin sensors, which were hydrated for 15 min before being used. The immobilized sensors were placed into the sample diluent buffer for 30 s to observe the dissociation of anti-PSA. The anti-PSA-immobilized sensor tips were placed into the sample diluent buffer (PBS) for 30 s to set the binding baseline. The sensors were then used to quantify biologically active PSA in the IL- and salt-rich phases of ABS. At least three independent ABS were prepared and 2 samples of each phase quantified.

The percentage extraction efficiency of PSA (${\text{EE}}_{{{\text{PSA}}}} {\text{\% }}$) is the percentage ratio between the amount of PSA in the IL-rich aqueous phase to that in the total mixture, and is defined according to Eq. (7):

7 $$EE_{{{\text{PSA}}}} \% = \frac{{w_{PSA}^{IL} }}{{w_{PSA}^{IL} + w_{PSA}^{Salt} }} \times 100$$

where $w_{PSA}^{IL}$ and $w_{PSA}^{Salt}$ are the total weight of PSA in the IL-rich and in the salt-rich aqueous phases, respectively. In all systems the top phase corresponds to the IL-rich phase whereas the bottom phase is mainly constituted by the salt and water.

The percentage recovery efficiency of PSA (*RY*_*PSA*_%) is the percentage ratio between the amount of PSA in the IL-rich aqueous phase to that in the initial PSA aqueous solution, and is defined according to Eq. (8):

8 $$RY_{{{\text{PSA}}}} \% = \frac{{w_{PSA}^{IL} }}{{w_{PSA}^{Total} }} \times 100$$

where $w_{PSA}^{IL}$ is the total weight of PSA in the IL-rich phase and $w_{PSA}^{Total}$ is the total weight of PSA in aqueous solution.

For the samples in which a concentration step was applied, PSA was quantified by SE-HLC. After a careful separation of the ABS phases, both phases were analysed by SE-HPLC. Each phase was diluted at a 1:9 (v:v) ratio in a phosphate buffer solution before injection. A Chromaster HPLC (VWR Hitachi) was used. The SE-HPLC was performed with an analytical column Shodex Protein KW- 802.5 (8 mm × 300 mm). A 100 mM phosphate buffer + NaCl 0.3 M was run isocratically with a flow rate of 0.5 mL.min^−1^. The column oven and autosampler temperatures were kept at 25 °C and at 10 °C, respectively. The injection volume was 25 μL. The wavelength was set at 280 nm using a DAD detector. The obtained chromatograms were treated and analysed using the OriginPro8 software. Equations (7) and (8) were applied to determine the extraction efficiency and recovery efficiency of the studied ABS for PSA.

### Lever-arm rule

The lever-arm rule was used to determine the weight percentages ratio of the coexisting phases in the respective phase diagrams for given mixture compositions. Several extractions were carried out at different compositions in the same TL, allowing to work with different concentration factors. A fixed and long TL was initially selected, and the lever-arm rule was used to determine the weight fraction of each phase-forming component (IL and K_3_C_6_H_5_O_7_) to be used in each extraction corresponding to a given concentration factor (CF). The CF is defined as the ability along the same TL to maintain the composition of each phase while varying only the volume and weight ratio of the phases. Several ternary mixtures were prepared within the biphasic region with the “theoretical” weight percentages of salt, IL and H_2_O/PSA, corresponding to CFs of 5, 20, 50, 100, 150, 200 and 250-fold. ABS were first prepared as a control without adding PSA, and once achieved the CF of 250-fold, the extractions were performed adding an aqueous solution of PSA at a concentration of 150 ng mL^−1^ (the cut-off value found in urine^13^). Each mixture was vigorously stirred, centrifuged for 10 min, and left to equilibrate at (25 ± 1)°C.

## Supplementary information

Supplementary file 1
