# Supplementary material for: Towards the differential diagnosis of prostate cancer by the pre-treatment of human urine using ionic liquids
Source: Sci Rep. 2020 Sep 10;10:14931. doi: 10.1038/s41598-020-71925-8 (PMC7483695; doi:10.1038/s41598-020-71925-8)
Supplement: Supplementary file 1 [file 41598_2020_71925_MOESM1_ESM.pdf]

Towards the differential diagnosis of prostate cancer by the pre-treatment of human urine using ionic liquids

Matheus M. Pereira[1], João D. Calixto[1], Ana C. A. Sousa[1], Bruno J. Pereira[2,3], Álvaro S. Lima[4], João A. P. Coutinho[1] and Mara G. Freire[1]

[1]CICECO - Aveiro Institute of Materials, Department of Chemistry, University of Aveiro, 3810-193 Aveiro, Portugal
[2]Faculty of Health Sciences, University of Beira Interior
6200-506 Covilhã, Portugal
[3]Instituto Português de Oncologia de Coimbra Francisco Gentil, 3000-075 Coimbra, Portugal
[4]Programa de Pós-Graduação em Engenharia de Processos, Universidade Tiradentes, Farolândia, CEP 49032-490 Aracaju, SE, Brazil
*E-mail: maragfreire@ua.pt

Supporting Information

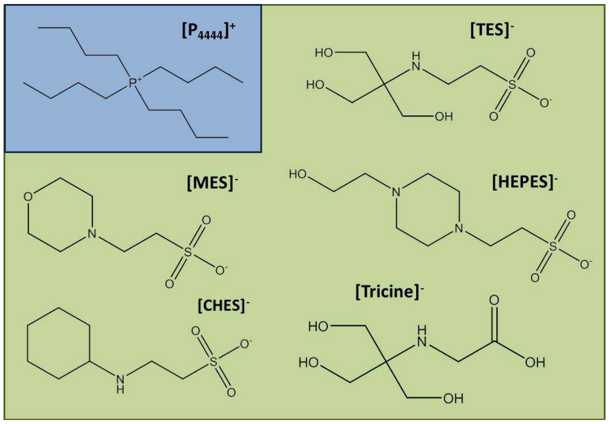

Figure S1. Chemical structures and abbreviations of the GB-ILs studied.

GB-ILs Characterization

[P_{4444}][MES]: From the MES buffer (48.5 mmol), this compound was obtained as a white solid. Water content < 0.05 wt%. 1H NMR (300 MHz, D2O/TSP): 3.63 (t, 4H), 2.99 (t, 2H), , 2.72 (t, 2H), 2.50 (t, 4H), 2.02 (m, 8H), 1.27-1.42 (m, 16H), 0.78 (t,12H,). 13C NMR (75.47 MHz, D2O/TSP): 68.80, 55.44, 55.11, 50.02, 26.28, 20.80, 20.16, 15.44.

[P_{4444}][TES]: From the TES buffer (45.1 mmol), this compound was obtained as a transparent viscous liquid. Water content < 0.05 wt%. 1H NMR (300 MHz, D2O/TSP): 3.31 (s, 6H), 2.82 (s, 2H), 2.57 (t, 2H), 2.10 (m, 8H), 1.29-1.49 (m,16H), 0.79 (t, 12H). 13C NMR (75.47 MHz, D2O/TSP): 60.65, 57.63, 51.58, 37.69, 26.28, 20.83, 20.17, 15.46.

[P_{4444}][CHES]: From the CHES buffer (47.2 mmol), this compound was obtained as a transparent viscous liquid. Water content <0.05 wt%. 1H NMR (300 MHz, D2O/TSP); 1H NMR (300 MHz, D2O/TSP); 2.95 (m, 2H), 2.38-2.47 (*m*, 1*H*), 2.02 (m, 8H), 1.28-1.45 (m, 16H,), 0.94 (m, 10H), 0.78 (t, 12H). 13C NMR (75.47 MHz, D2O/TSP): 63.37, 63.09, 53.42, 39.56, 26.28, 26.08, 25.60, 20.30, 20.15, 15.43.

[P_{4444}][HEPES]: From the HEPES buffer (44.3 mmol), this compound was obtained as a yellowish white solid. Water content < 0.05 wt%. 1H NMR (300 MHz, D2O/TSP): 3.59 (t,4H), 2.97 (t, 2H), 2.94 (t, 2H), 2.67 (t, 2H), 2.46 (t, 8H), 2.03 (m, 8H), 1.28-1.45 (m, 16H), 0.79 (t, 12H). 13C NMR (75.47 MHz, D2O/TSP): 61.59, 60.87, 55.02, 54.78, 54.19, 50.39, 26.29, 20.80, 20.16, 15.44.

[P_{4444}][Tricine]: From the Tricine buffer (50.3 mmol), this compound was obtained as white solid. Water content < 0.05 wt%. 1H NMR (300 MHz, D2O/TSP): 3.37 (s, 6H), 3.12 (s, 2H), 2.00 (m, 8H), 1.27-1.44 (m, 16H), 0.77 (t. 12H). 13C NMR (75.47 MHz, D2O/TSP): 182.71, 63.12, 62.97, 47.68, 26.28, 20.79, 20.15, 15.42.

Table S1. Correlation parameters of Equation (1) used to describe the experimental binodal data at 25°C.

IL + salt + water					
**IL**	**Salt**	***A* ± *σ***	***B* ± *σ***	**10^5 (*C* ± *σ*)**	**R^2**
$[P_{4444}]$[MES]	$K_3C_6H_5O_7$	98.33 ± 0.54	-0.327 ± 0.022	3.96 ± 0.06	0.9996
$[P_{4444}]$[CHES]		111.92 ± 2.49	-0.450 ± 0.009	10.50 ± 0.44	0.9929
$[P_{4444}]$[HEPES]		105.17 ± 1.19	-0.295 ± 4.257	2.78 ± 0.08	0.9978
$[P_{4444}]$[Tricine]		105.15 ± 5.10	-0.248 ± 0.016	1.71 ± 0.02	0.9992
$[P_{4444}]$[TES]		99.63 ± 0.51	-0.356 ± 0.007	5.40 ± 0.29	0.9971

Table S2. Experimental TLs and TLLs of the ABS composed of IL + $K_3C_6H_5O_7$ + H_2O at 25°C.

Weight fraction composition / (wt %)							
IL + $K_3C_6H_5O_7$ + water							
**IL**	**$[IL]_{IL}$**	**$[salt]_{IL}$**	**$[IL]_M$**	**$[salt]_M$**	**$[IL]_{salt}$**	**$[salt]_{salt}$**	**TLL**
$[P_{4444}]$[MES]	65.71	1.51	29.42	20.48	2.89	34.36	70.89
	90.81	0.06	29.36	30.33	0.37	44.12	100.60
	94.29	0.01	30.21	39.87	$0.27.10^{-3}$	58.64	111.04
$[P_{4444}]$[CHES]	67.45	1.26	29.70	19.83	0.11	34.38	75.04
	75.58	0.76	29.64	30.25	0.17×10^{-4}	49.27	89.81
	93.56	0.16	30.11	39.95	0.18×10^{-8}	58.83	110.44
$[P_{4444}]$[HEPES]	57.51	4.14	29.89	20.14	6.59	33.63	57.80
	83.11	0.63	30.24	29.49	0.95	46.00	93.84
	90.97	0.24	29.72	40.13	0.03	59.46	108.52
$[P_{4444}]$[Tricine]	52.11	7.75	30.08	24.73	4.42	44.50	60.20
	70.02	2.64	30.45	29.71	2.53	48.78	81.75
	94.31	0.19	29.78	40.11	0.54	58.20	110.26
$[P_{4444}]$[TES]	50.61	5.97	30.28	19.70	0.39	39.87	60.59
	93.58	0.05	30.69	29.89	0.08	44.32	103.45
	96.00	0.02	30.43	39.48	0.11×10^{-3}	57.80	112.05

Table S3. Experimental weight fraction data for the ABS composed of IL (1) + $K_3C_6H_5O_7$ (2) + H_2O (3) at 25 °C and atmospheric pressure.

[P_{4444}][TES]		[P_{4444}][MES]		[P_{4444}][HEPES]	
100 w_1	100 w_2	100 w_1	100 w_2	100 w_1	100 w_2
76.71	0.96	62.37	1.95	79.05	1.21
70.87	1.36	55.07	3.07	57.38	3.82
68.27	2.17	46.15	5.21	54.39	4.36
65.19	2.44	38.99	7.55	52.99	4.88
61.73	2.92	35.53	9.45	51.00	5.21
59.35	3.82	32.17	10.43	49.78	5.79
57.40	4.38	29.24	12.18	48.55	6.45
55.12	4.67	25.01	14.22	46.85	6.80
51.18	5.62	24.37	14.94	45.84	7.21
48.08	6.76	23.45	15.59	44.92	7.54
45.45	7.58	22.73	15.99	43.94	8.03
44.36	8.01	22.10	16.27	43.08	8.33
43.30	8.31	21.05	17.21	42.24	8.63
41.47	9.27	20.19	17.49	41.06	9.46
39.38	10.12	19.88	17.71	40.29	9.76
37.71	10.70	19.30	18.08	39.61	9.99
36.76	11.09	18.82	18.32	38.78	10.41
35.71	11.69	18.48	18.78	38.09	10.79
34.46	12.49	17.96	19.05	37.15	11.51
33.18	12.85	17.56	19.34	36.51	11.87
32.30	13.27	17.16	19.51	35.92	12.10
31.34	13.85	16.73	19.90	35.09	12.68
30.81	13.98	16.30	20.01	34.58	12.88
29.88	14.51	15.93	20.21	33.99	13.09
29.46	14.56	15.77	20.58	33.20	13.63
28.66	14.96	15.05	21.16	32.72	13.81
27.96	15.39	14.78	21.34	31.98	14.39
27.22	15.82	14.47	21.49	31.54	14.60
26.42	15.92	14.02	21.86	31.09	14.79
25.57	16.33	13.60	22.21	30.50	15.29
24.21	16.84	13.35	22.44	30.07	15.47
23.85	16.93	13.12	22.55	29.44	15.97
23.30	17.33	12.86	22.75	29.03	16.18
22.99	17.35	12.62	22.89	28.48	16.60
22.38	17.80	12.33	23.10	28.11	16.77
21.60	18.21	12.13	23.25	27.54	17.25
21.33	18.26	11.94	23.41	27.16	17.43
20.90	18.58	11.64	23.79	26.77	17.63
20.47	18.89	11.41	23.87	26.26	18.10
20.22	18.89	11.19	24.14	25.91	18.23
19.77	19.19	10.81	24.41	25.55	18.44
19.25	19.37	10.64	24.57	25.09	18.84
18.93	19.56	10.26	25.32	24.76	19.04

18.65	19.78	10.05	25.33	24.43	19.19
18.28	20.02	9.90	25.44	24.13	19.35
17.65	20.34	9.76	25.58	23.85	19.47
17.44	20.36	9.49	25.92	23.56	19.66
17.12	20.62	9.35	26.10	23.14	20.09
16.97	20.76			22.88	20.22
16.55	21.14			22.60	20.41
16.16	21.25			22.37	20.51
15.95	21.33			22.09	20.67
15.78	21.37			21.74	21.01
15.56	21.32			21.49	21.16
15.14	21.59			21.23	21.33
14.97	21.66			20.99	21.51
14.68	21.95			20.76	21.65
14.35	22.09			20.50	21.80
14.21	22.12			20.17	22.17
14.06	22.17			19.95	22.32
13.81	22.43			19.74	22.44
13.46	22.70			19.53	22.58
13.30	22.75			19.33	22.70
13.00	23.11			19.13	22.81
12.87	23.18			18.93	22.94
12.66	23.37			18.74	23.05
12.36	23.56			18.56	23.13
12.12	23.69			18.37	23.28
11.86	23.81			18.18	23.40
11.66	23.92			17.99	23.56
11.44	24.04			17.80	23.71
11.15	24.30			17.64	23.81
10.94	24.38			17.47	23.90
10.68	24.58			17.31	23.99
10.50	24.64			17.06	24.27
10.28	24.80			16.91	24.34
9.98	25.04			16.76	24.43
				16.62	24.49
				16.46	24.55
				16.31	24.67
				16.16	24.83
				16.01	24.98
				15.89	25.08
				15.75	25.17
				15.61	25.27
				15.44	25.35
				15.25	25.43
				14.96	25.57
				14.78	25.57
				14.66	25.64

14.54	25.73
14.43	25.80
14.33	25.87
14.22	25.96
14.11	26.08
14.01	26.14
13.90	26.24
13.68	26.44
13.44	26.52
13.33	26.61
13.12	26.95
13.02	27.04
12.93	27.10
12.82	27.18
12.73	27.26
12.64	27.31
12.56	27.38
12.45	27.48
12.35	27.56
12.26	27.64
12.18	27.71
12.04	27.55
11.91	27.53
11.83	27.64
11.74	27.74
11.66	27.79
11.57	27.79
11.44	28.00
11.36	28.06
11.21	28.19
11.13	28.24
11.06	28.32
10.98	28.36
10.90	28.43
10.82	28.50
10.75	28.55
10.69	28.59
10.56	28.68
10.45	28.84
10.39	28.88
10.31	28.92
10.17	29.06
10.10	29.11
10.00	29.20
9.93	29.24
9.87	29.29
9.77	29.42

9.71	29.47
9.65	29.52
9.60	29.55
9.51	29.73
9.39	29.79
9.29	29.88
9.13	29.82
9.01	29.84
9.08	30.39
8.39	31.06
7.78	31.65
7.40	31.77
7.01	31.97
6.68	32.26
6.31	32.71
5.94	33.24
5.73	33.55
5.43	34.05
5.19	34.18
4.90	34.49
4.65	34.80
4.42	35.07
4.20	35.30
3.96	35.32
3.90	35.14
3.47	35.48
3.24	35.97
3.03	36.46
2.84	36.98
2.67	37.33
2.53	37.67
2.36	38.17
2.18	38.61
2.03	39.14
1.87	39.49
1.71	39.92
1.55	40.36
1.39	40.79
1.23	41.22
1.07	41.65
0.90	42.08
0.74	42.51
0.58	42.94
0.42	43.38
0.26	43.81
0.10	44.24

Table S4. Experimental weight fraction data for the ABS composed of IL (1) + $K_3C_6H_5O_7$ (2) + H_2O (3) at 25 °C and atmospheric pressure.

[P_{4444}][CHES]		[P_{4444}][Tricine]	
100 w_1	100 w_2	100 w_1	100 w_2
44.76	3.92	77.51	1.40
41.71	4.74	69.04	3.28
39.33	5.34	60.39	4.60
37.55	5.83	45.31	10.91
36.22	6.34	43.30	12.04
34.48	6.67	41.79	12.71
33.48	7.04	40.77	13.40
32.32	7.47	39.41	13.86
30.98	7.83	38.48	14.52
29.73	8.17	37.52	15.07
28.73	8.39	36.64	15.63
27.94	8.79	35.83	16.16
27.39	9.18	35.08	16.64
26.69	9.53	34.32	17.15
25.86	9.67	33.65	17.61
25.25	10.02	32.93	18.09
24.53	10.24	32.27	18.52
23.96	10.60	31.59	18.97
23.32	10.72	30.98	19.39
22.79	10.83	30.45	19.68
22.46	11.16	29.86	20.06
21.96	11.28	29.33	20.41
21.47	11.46	28.80	20.78
21.06	11.73	27.97	21.61
20.53	11.92	27.50	21.99
20.03	12.05	27.05	22.29
19.69	12.25	26.61	22.58
19.27	12.36	26.18	22.80
18.95	12.57	25.52	23.47
18.58	12.67	25.11	23.75
18.27	12.86	24.71	23.98
17.91	12.90	24.29	24.15
17.61	13.14	23.95	24.37
17.24	13.19	23.41	24.94
16.95	13.38	23.08	25.13
16.60	13.53	22.78	25.31
16.27	13.70	21.98	25.95
15.94	13.83	21.69	26.17
15.64	13.97	21.24	26.63
15.35	14.12	20.99	26.78
15.08	14.19	20.71	26.95
14.87	14.13	20.26	27.41
14.62	14.20	20.04	27.47

14.36	14.23	19.66	27.85
14.17	14.41	19.44	28.02
13.93	14.57	19.21	28.17
13.71	14.61	18.90	28.45
13.49	14.73	18.61	28.74
13.32	14.86	18.30	29.02
13.10	14.96	18.11	29.14
12.89	15.07	17.78	29.48
12.69	15.11	17.58	29.57
12.53	15.23	17.41	29.75
12.36	15.28	17.19	29.88
12.18	15.41	16.93	30.15
11.96	15.35	16.67	30.38
11.87	15.52	16.51	30.52
11.73	15.61	16.25	30.77
11.61	15.71	16.09	30.87
11.47	15.78	15.86	31.06
11.33	15.81	15.61	31.28
11.22	15.89	15.36	31.53
11.11	15.94	15.22	31.58
11.01	16.05	14.99	31.80
10.89	16.07	14.77	32.00
10.78	16.18	14.54	32.24
10.59	16.16	14.27	32.41
10.50	16.22	14.09	32.60
10.40	16.32	13.97	32.64
10.30	16.38	13.78	32.85
10.18	16.43	13.64	32.97
10.05	16.65	13.47	33.15
9.90	16.63	13.27	33.23
9.81	16.70	13.10	33.42
9.73	16.76	12.88	33.71
9.65	16.83	12.77	33.76
9.55	16.88	12.49	33.98
9.46	16.96	12.03	34.02
9.37	16.94	11.87	34.18
9.28	17.00	11.71	34.36
9.17	17.17	11.58	34.49
9.04	17.35	11.43	34.66
8.88	17.45	11.31	34.79
7.88	19.13	11.16	34.83
7.59	19.32	11.03	35.00
7.24	19.86	10.89	35.17
6.97	19.88	10.81	35.17
6.67	20.18	10.70	35.31
6.39	20.24	10.46	35.57
6.16	20.70	10.62	35.21

5.89	20.61	10.23	35.74
5.59	21.35	9.84	36.26
5.27	21.22	9.45	36.79
5.05	21.46	9.06	37.32
4.85	21.89	8.68	37.85
4.64	22.17	8.29	38.37
4.44	22.21	7.90	38.91
4.28	22.48	7.51	39.43
4.15	22.83	7.12	39.95
3.98	23.02	6.72	40.44
3.80	23.28	6.36	41.05
3.67	23.42	5.95	41.44
3.52	23.64	5.55	42.03
3.42	23.80	5.36	42.28
3.30	23.90	5.07	42.55
3.22	24.19	4.87	42.87
3.11	24.21	4.75	42.71
3.00	24.53	4.50	43.09
2.90	25.13	4.30	43.28
2.79	25.33	4.08	43.58
2.71	25.53	3.86	43.86
2.62	25.68	3.73	43.88
2.62	24.71	3.61	43.81
2.45	24.16	3.42	44.21
2.38	24.30	3.28	44.45
2.31	24.53	3.13	44.73
2.25	24.62	2.98	45.05
2.16	24.77	2.86	45.34
2.08	25.04	2.74	45.59
2.00	25.34	2.59	45.88
1.91	25.76	2.48	46.17
1.83	25.92	2.33	46.53
1.75	26.28	2.23	46.67
1.67	26.45	2.13	46.91
1.60	26.74	1.99	47.40
1.52	26.98	1.89	47.13
1.46	27.28	1.74	47.23
1.39	27.59		
1.31	27.82		
1.23	28.19		
1.17	28.53		
1.10	28.85		
1.04	29.09		
0.99	29.39		

Table S5. Extraction efficiency (*EE_{PSA}*%) and recovery efficiencies (*RY_{PSA}*%) of PSA at 25° C in the ABS composed of ILs and $K_3C_6H_5O_7$.

IL	Weight fraction composition / (wt %)		EE_{PSA}%	RY_{PSA}%
	[IL]IL	[salt]IL		
[P4444][MES]	30.35 ± 0.15	30.84 ± 0.53	100	96.32 ± 1.05
	30.01 ± 0.04	40.03 ± 0.14	100	95.97 ± 0.96
[P4444][CHES]	30.13 ± 0.25	30.54 ± 0.63	100	97.96 ± 0.90
	29.94 ± 0.13	40.12 ± 0.57	100	98.53 ± 1.13
[P4444][HEPES]	30.16 ± 0.35	30.44 ± 0.29	100	9.88 ± 1.79
	30.09 ± 0.34	39.97 ± 0.27	100	10.51 ± 1.12
[P4444][Tricine]	30.90 ± 0.28	30.70 ± 0.31	100	6.58 ± 1.09
	30.37 ± 0.17	39.98 ± 0.07	100	7.08 ± 0.45
[P4444][TES]	29.95 ± 0.24	30.08 ± 0.69	100	31.77 ± 1.68
	30.63 ± 0.37	39.85 ± 0.17	100	32.01 ± 0.96

Table S6. Experimental data for the CF of the systems composed of [P_{4444}][GB] + $K_3C_6H_5O_7$ + water at 25°C.

[P_{4444}][MES]							
**CF**	5	20	50	100	150	200	250
**w IL / g**	0.1538	0.8746	0.9034	0.4669	0.608	0.3206	0.2578
**w Salt/ g**	0.4907	11.1844	28.8004	29.0330	58.2693	40.8537	40.8927
**w H_2O/ g**	0.3556	7.9404	20.3765	20.5313	41.1231	28.8267	28.8521
**w Top/ g**	0.0783	0.4023	0.4108	0.2064	0.2603	0.1476	0.1166
**w Bottom/ g**	0.8099	18.7884	48.9843	46.2209	98.9366	69.2375	69.1359
**Real *CF***	4.54	19.74	49.60	99.47	157.98	195.30	247.45

[P_{4444}][CHES]							
**CF**	5	20	50	100	150	200	250
**w IL / g**	0.1546	0.8718	0.896	0.4543	0.3079	0.317	0.2541
**w Salt/ g**	0.4918	11.2273	28.8647	29.1384	29.2327	40.9848	41.0242
**w H_2O/ g**	0.3553	7.9012	20.2728	20.4156	20.4793	28.7043	28.7221
**w Top/ g**	0.0855	0.4322	0.3842	0.2101	0.1319	0.1406	0.1174
**w Bottom/ g**	0.8246	17.8944	48.8542	49.2884	49.1391	68.4552	69.6864
**Real *CF***	4.16	18.28	52.77	97.17	155.26	204.16	244.65

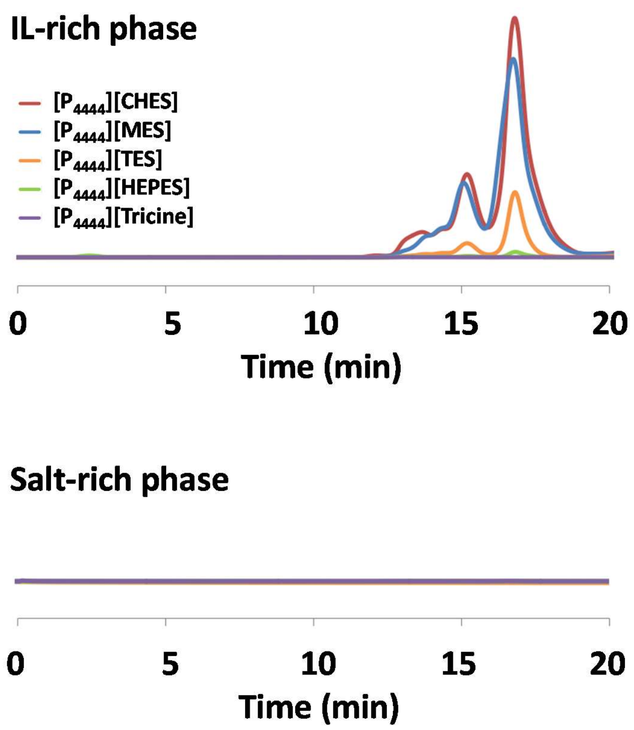

Figure S2. SE-HPLC profiles of ABS composed of $[P_{4444}][GB]$ + salt + PSA aqueous solution (150 ng.mL^{-1}).
